# Which Specialized Metabolites Does the Native Subantarctic Gastropod *Notodiscus hookeri* Extract from the Consumption of the Lichens *Usnea taylorii* and *Pseudocyphellaria crocata*?

**DOI:** 10.3390/molecules22030425

**Published:** 2017-03-08

**Authors:** Alice Gadea, Pierre Le Pogam, Grichka Biver, Joël Boustie, Anne-Cécile Le Lamer, Françoise Le Dévéhat, Maryvonne Charrier

**Affiliations:** 1 Université Bretagne–Loire, Université de Rennes 1, UMR CNRS 6553 (ECOBIO), 263 Avenue du Général Leclerc, 35042 Rennes CEDEX, France; alice.gadea@univ-rennes1.fr (A.G.); grichka.biver1@gmail.com (G.B.); 2 Université Bretagne–Loire, Université de Rennes 1, UMR CNRS 6226 (ISCR), 2 Avenue du Professeur Léon Bernard, 35043 Rennes CEDEX, France; pierre.lepogam.alluard@gmail.com (P.L.P.); joel.boustie@univ-rennes1.fr (J.B.); anne-cecile.le-lamer@univ-tlse3.fr (A.-C.L.L.); francoise.le-devehat@univ-rennes1.fr (F.L.D.); 3 Université Bretagne–Loire, Université de Rennes 1, UMR CNRS 6164 (IETR), 263 Avenue du Général Leclerc, 35042 Rennes CEDEX, France; 4 Université Midi–Pyrénées, Université Paul Sabatier Toulouse 3, 118 Route de Narbonne, 31062 Toulouse CEDEX, France

**Keywords:** lichens, snails, chemical ecology, Crozet Archipelago, *Notodiscus hookeri*, *Usnea taylorii*, *Pseudocyphellaria crocata*

## Abstract

*Notodiscus hookeri* is the only representative of terrestrial gastropods on Possession Island and exclusively feeds on lichens. The known toxicity of various lichen metabolites to plant-eating invertebrates led us to propose that *N. hookeri* evolved means to protect itself from their adverse effects. To validate this assumption, the current study focused on the consumption of two lichen species: *Usnea taylorii* and *Pseudocyphellaria crocata*. A controlled feeding experiment was designed to understand how the snail copes with the unpalatable and/or toxic compounds produced by these lichen species. The occurrence of two snail ecophenotypes, represented by a mineral shell and an organic shell, led to address the question of a metabolic response specific to the phenotype. Snails were fed for two months with one of these lichens and the chemical profiles of biological samples of *N. hookeri* (i.e., crop, digestive gland, intestine, and feces) were established by HPLC-DAD-MS and compared to that of the lichens. *N. hookeri* appears as a generalist lichen feeder able to consume toxic metabolite-containing lichens, independently of the ecophenotype. The digestive gland did not sequester lichen metabolites. The snail metabolism might be based on four non-exclusive processes according to the concerned metabolites (avoidance, passive transport, hydrolysis, and excretion).

## 1. Introduction

Lichens are fascinating self-sustaining partnerships consisting of a fungus and a photobiont partner (in most cases, green algae are sometimes replaced or accompanied by cyanobacteria). Owing to their nutritional autonomy, lichens tend to dominate nutrient-poor, cold and/or dry environments. As such, they stand for the major form of vegetation in polar and alpine regions and in particularly harsh climate zones [1] in which they often represent the dominant form of multicellular life [2]. As these extreme habitats have few and low-quantity fodder plants for herbivores, the lichens will represent a major trophic resource [3]. The extreme resiliency of lichens partly relies on the synthesis of numerous unique specialized metabolites displaying a wide array of biological activities affecting both their biotic and abiotic interactions [4]. As to the former point, several publications established that the lichen diet of various insects was species, and especially lichen-chemistry, selective. For instance, studies carried out on the polyphagous coleopteran *Lasioderma serricorne* highlighted a negative correlation between various lichen substances and grazing phenomena [5]. In a similar way, the land snail *Cantareus aspersa* and the slug *Limax* sp. were shown to prefer *Peltigera* lichens lacking secondary metabolites [6]. Due to the extracellular distribution of lichen specialized metabolites, these substances can be non-destructively extracted with acetone through the so-called acetone-rinsing technique without harming the lichen symbionts, offering a powerful experimental setup to evaluate the deterrent functions of lichen metabolites against plant-eating species [7]. Using such experimental design, several authors emphasized deterrence and/or toxicity of either pure substances of various structural classes or crude lichen extracts towards insect larvae, moth larvae, bank voles, slugs, and snails (for review, see [3]).

Among plants, dietary preferences of herbivores are obviously related to chemical cues with various structural families being found to deter damages induced by terrestrial snails. Some such examples include glucosinolates [8], terpenes [9], and alkaloids [10]. However, some gastropods are able to feed on resources containing toxic secondary metabolites. Some poisonous molecules should be degraded in the gut lumen prior to being absorbed or detoxified by microsomal cytochrome P450 enzymes contained in the digestive gland [11]. This mechanism of detoxification was evidenced for alkaloids in the slug *Arion lusitanicus* [12].

Likewise, one can assume that the consumption of toxic lichen compounds might result in defensive strategies (i.e., sequestration of toxic compounds, biotransformation by conjugation to polar metabolites, etc.) by lichenophagic species to overcome potential intoxications. Even though data regarding lichenophagic snail species are scarce, Hesbacher and co-workers highlighted the specific uptake of two lichen polyphenols—atranorin and parietin—by the snails *Balea perversa*, *Chondrina clienta*, and *Helicigona lapicida* whereas usnic acid and α-collatolic acid could not be detected in the soft bodies of the gastropods. Most interestingly, in the ovoviviparous species *Balea perversa*, the sequestrated parietin could be transmitted to the offspring in the reproductive tract [13].

To advance the understanding of the chemical ecology underlying lichen/snails interactions, *Notodiscus hookeri*, a subantarctic snail harvested in Possession Island, appeared as a relevant model organism. The Crozet Archipelago is located in the subantarctic region in the South Indian Ocean province. Among these islands, Possession Island is a strato-volcano island displaying a mountainous topography (highest peak: Pic du Mascarin at 934 m) extending over a short distance (18 km × 15 km) [14]. This topology is associated with a wet and windy climate [15]. As a consequence of these harsh environmental conditions, the Subantarctic islands display a low terrestrial biodiversity, resulting in relatively simple ecosystems [16,17]. For instance, native vascular plants account for less than 30 species, while about 50 native invertebrate species have been described so far [18]. Among them *Notodiscus hookeri* (Charopidae) is the only terrestrial gastropod species found in the South Indian Ocean Islands and archipelagos [15]. This litter-dwelling species includes two ecophenotypes, one of which—called mineral phenotype (MP)—has available calcium in soil and moist stones covered by vegetation, while the other, known as organic phenotype (OP), is widespread in fell-field areas dominated by lichen species but deprived of exchangeable calcium in soil [15] MP shells are twice as thick as OP shells (72 µm versus 35 µm in mean) and OP snails exhibit a predominantly organic skeleton made of glycine-rich proteins [15]. With a higher energetic cost being involved in protein production compared to calcification [19], we expected OP snails to manage more efficiently than MP snails’ toxic compounds in lichens in order to extract the highest caloric content, at least on species selected in their environment. Therefore, due to these environmental and phenotypic specificities, we hypothesized that *N. hookeri* developed detoxification mechanisms by either degradation and/or elimination of toxic secondary metabolites so that it can benefit from its unique trophic resource. During the course of this study, the consumption of two lichen species was considered, *Usnea taylorii* and *Pseudocyphellaria crocata,* which occur abundantly in the fell-fields and the coastal plains, respectively (Figure S1). *N. hookeri* can feed on both of them, independently from their occurrence in the harvest site of the snails. Nevertheless, assuming that the detoxification strategies implemented by *N. hookeri* might depend on its previous consumption of these lichens, we hypothesized that OP snails would better cope with the toxic metabolites of *U. taylorii* metabolites and MP snails those of *P. crocata*. A further interest of these lichen species is that they differ by the nature of their photobionts: *U. taylorii* contains trebouxioid green algae whereas *P. crocata* incorporates *Nostoc* cyanobacteria. Additionally, both are known to contain secondary metabolites that exert documented deterrent effects and/or toxicity towards invertebrate phytophages [3].

In this study, we investigated the fate of lichen metabolites after their consumption by *Notodiscus hookeri*. For this purpose, the chemical profiles of *U. taylorii* and *P. crocata* were assessed by HPLC-DAD-MS. Their major compounds were quantified and subsequently compared with the content of the crop, digestive gland, intestine, and feces of the snails (Figure S2) fed with either one of the aforementioned lichen species. Finally, the diverse strategies implemented by *N. hookeri* to circumvent the toxicity of their lichen metabolites are discussed.

## 2. Results

### 2.1. Chemical Profiling of the Lichens and Quantification of Their Major Compounds

The chemical profiles of *U. taylorii* and *P. crocata* were established by HPLC-DAD-ESI-MS of their acetone extracts. Lichen metabolites were identified in the extracts based on comparison with retention times, UV/Vis, and MS spectra of pure compounds. Linear equations developed on the basis of the calibration curves were used to determine the percentage of the major compounds of these lichens.

#### 2.1.1. *Usnea taylorii*

**Figure 1:**
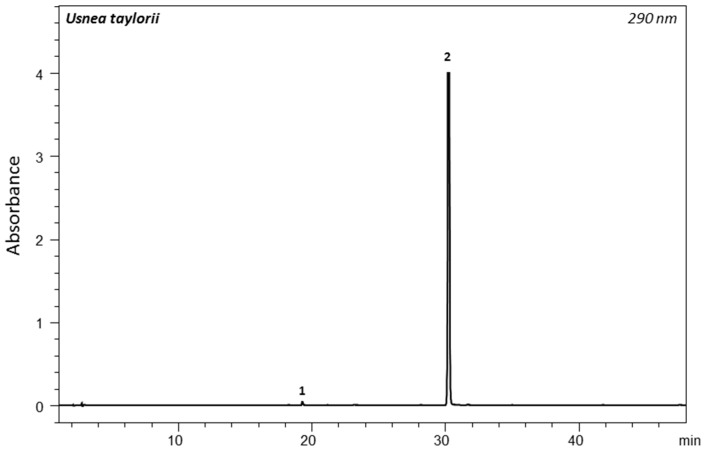
PDA chromatogram at 290 nm of *U. taylorii* acetone extract. Identified compounds are: (**1**) fumarprotocetraric acid (traces) and (**2**) usnic acid.

*Usnea taylorii* appears to be rather poor in secondary metabolites as its extraction yield is of 0.8% ± 0.2%. The PDA chromatogram of the acetone extract of *U. taylorii* is provided in Figure 1.

The chemistry of *Usnea taylorii* is heavily dominated by the dibenzofuran-related usnic acid (**2**, Rt = 30 min, *m*/*z* = 343 [M − H]^−^, 328 and 259) (Figure S3). Its concentration ranged between 2.54 and 4.92 mg/g Dry Weight (DW) of lichen for a mean value of 3.75 ± 1.18 mg/g DW, which stands for 0.4% of the dried lichen material. Fumarprotocetraric acid could also be detected (**1**, Rt = 19.6 min, *m*/*z* = 471 [M − H]^−^ and 355) (Figure S4).

#### 2.1.2. *Pseudocyphellaria crocata*

**Figure 2:**
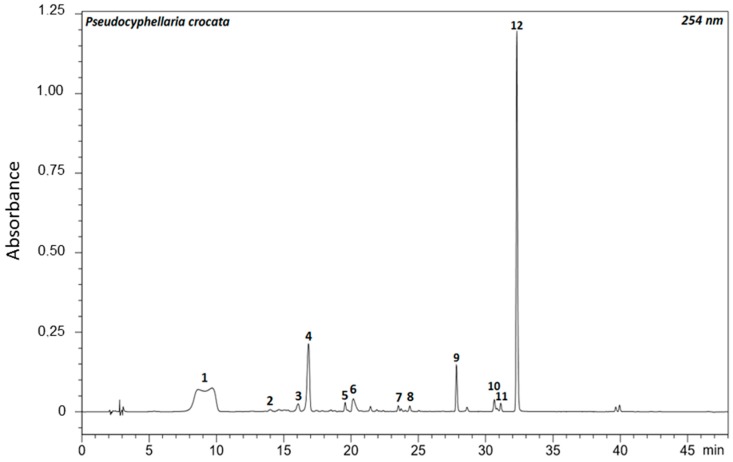
PDA chromatogram at 254 nm of *P. crocata* acetone extract. Identified compounds are (**1**) constictic acid; (**2**) cryptostictic acid; (**3**) Pc3; (4) stictic acid; (**5**) norstictic acid; (**6**) pulvinic acid; (**7**) gyrophoric acid; (**8**) pulvinamide; (**9**) 4-*O*-methylgyrophoric acid; (**10**) calycin; (**11**) Pc11; and (**12**) tenuiorin.

*Pseudocyphellaria* sp. displays a complex chemistry of secondary metabolites encompassing a wide structural diversity, which is in line with a higher extraction yield of 5.3% ± 1.2%. Twelve main compounds falling into three structural series of compounds could be evidenced, consistent with the literature data. The UV chromatogram recorded at 254 nm is displayed below (Figure 2).

At first, the polar metabolites **1**, **2**, **4** and **5** could be identified as depsidones in a straightforward manner owing to their prevalent deprotonated molecules (Figure S9). This led to identifying **1** as constictic acid (Rt = 9 min; *m*/*z* = 401 [M − H]^−^), **2** as cryptostictic acid (Rt = 14.3 min; *m*/*z* = 387 [M − H]^−^), **4** as stictic acid (Rt = 16.7 min; *m*/*z* = 385 [M − H]^−^), and **5** as norstictic acid (Rt = 19.3 min; *m*/*z* = 371 [M − H]^−^) (Figure S5). Compound **3**, named Pc3 (Rt = 16 min; *m*/*z* = 443, 387, 339) did not match with any previous report from *P. crocata* and could not be identified (Figure S6). The HPLC-DAD-MS analysis also revealed the occurrence of three tridepsides: gyrophoric acid (**7**, Rt = 23.5 min; *m*/*z* 149, 167, 317, 468 and 489), 4-*O*-methylgyrophoric acid (**9**, Rt = 27.8 min; *m*/*z* = 149, 167, 331, 481 [M − H]^−^), and tenuiorin (**12**, Rt = 32.5 min; *m*/*z* = 149, 167, 331, 495 [M − H]^−^) (Figures S7 and S8).

At last, 254 nm UV-detection revealed three more peaks that were tentatively identified as pulvinic acid derivatives with pulvinic acid (**6**, Rt = 20.3 min, *m*/*z* = 307 [M − H]^−^), pulvinamide (**8**, Rt = 24.5 min, *m*/*z* = 306 [M − H]^−^), and calycin (**10**, Rt = 30.5 min, *m*/*z* = 305 [M − H]^−^) (Figure S9). UV-detection at 419 nm enabled a more sensitive detection of these signals as described elsewhere [20]. The 419 nm PDA chromatogram is displayed in the Supplementary Materials (Figure S10).

Compound **11** (Pc11) did not ionize under the mass spectrometric conditions used in this study, precluding its structural assignment.

One representative of each structural class was then quantified. Tenuiorin appeared to be the main metabolite of *P. crocata* with a concentration of 18.01 ± 6.54 mg/g DW lichen followed by stictic acid 4.09 ± 1.06 mg/g DW lichen and calycin 1.17 ± 0.37 mg/g DW lichen.

**Figure 3:**
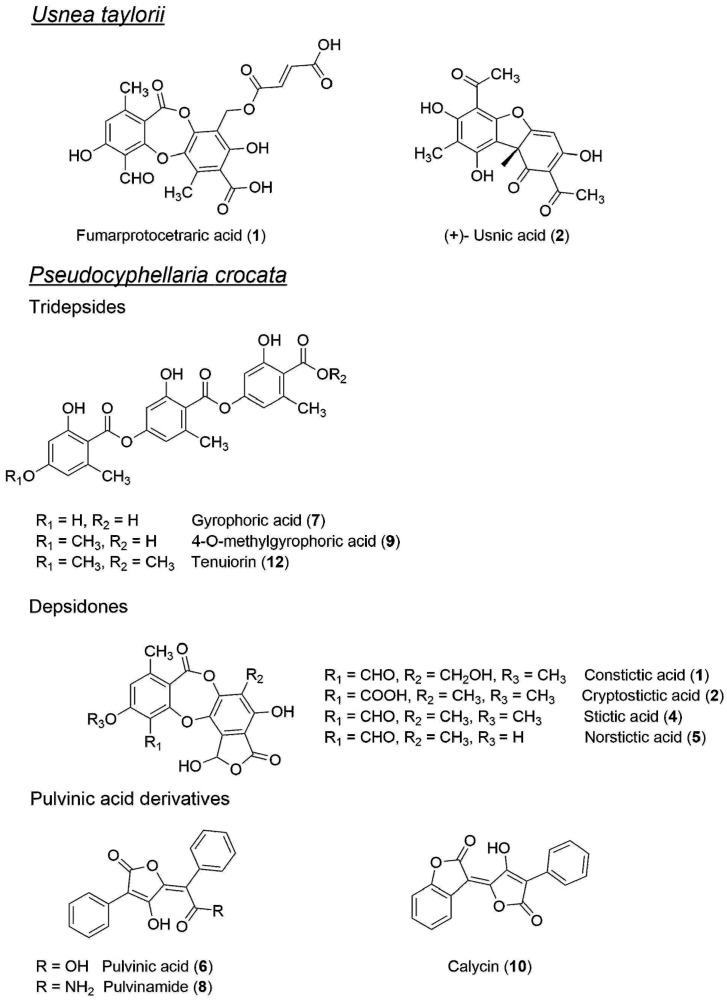
Structures of the compounds identified in *Usnea taylorii* and *Pseudocyphellaria crocata* collected on Possession Island (Crozet Archipelago).

The molecular formulae of the compounds identified from *Usnea taylorii* and *Pseudocyphellaria crocata* during the course of this study are collated in Figure 3.

### 2.2. Chemical Profiling of the Biological Tissues and Quantitation of Their Major Compounds

A first outcome is that higher extraction yields from biological tissues were obtained from snails fed with *P. crocata* compared with those having received *U. taylorii* (13.1% ± 5.4% vs. 8.5% ± 0.6%; 14% ± 5.6% vs. 9.6% ± 2.2%; 0.4% ± 0.2% vs. 0.3% ± 0.1% and 5.7% ± 1.3% vs. 3.4% ± 1.3% for crops, intestines, digestive glands, and feces, respectively).

#### 2.2.1. Products of the Digestion of *Usnea taylorii*

**Figure 4:**
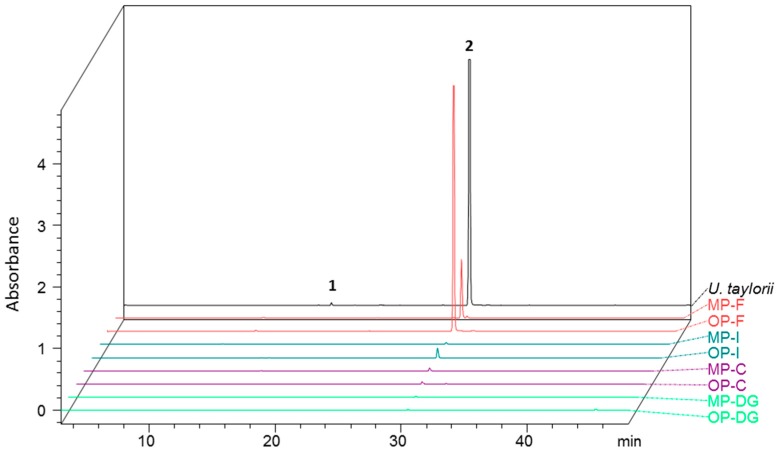
PDA chromatogram at 290 nm of acetone extracts of *N. hookeri* digestive gland (DG, green curves), crops (C, purple curves), intestines (I, blue curves), and feces (F, red curves) after being fed with *U. taylorii* (black curve). These profiles are compared according to their phenotype (MP = mineral, PBAS sample; OP = organic, MAS sample).

Two compounds were detected in *U. taylorii* and snail gut compartments (Figure 4). The lichen was characterized by usnic acid and traces of fumarprotocetraric, but only the former was found in the snail guts. Usnic acid was more abundant in the feces (mean values mg/g DW lichen ± Sd = 5.70 ± 2.21 in MP snails; 11.31 ± 2.28 in OP snails), compared to the soft bodies of the snails where the levels remained below 1.40 mg/g DW. The concentrations in the feces were not statistically different between ecophenotypes (*t*-test, *t* = −2.050, *p* = 0.119). It also appeared that the concentration of this dibenzofuran derivative was very low (<0.004 mg/g DW) in the digestive glands, whatever the snail ecophenotype. *U. taylorii*-fed snails excreted two to three-fold more usnic acid than the level found in the lichen (mean value ± Sd = 3.75 ± 1.18 mg/g) according to the ANOVA result (F_2,9_ = 4.429; *p* = 0.046). This difference was significant between OP snails and lichen content (Tukey test, *p* = 0.044), while usnic acid values did not differ statistically between MP snails and lichen content (Tukey test, *p* = 0.747).

#### 2.2.2. Products of the Digestion of *Pseudocyphellaria crocata*

**Figure 5:**
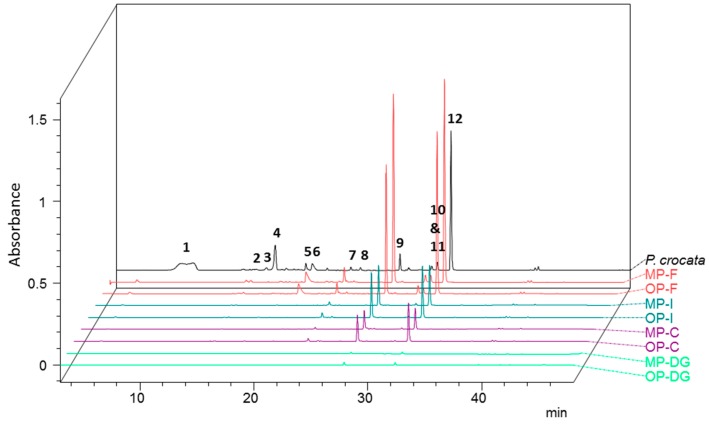
PDA chromatogram at 254 nm of acetone extracts of *N. hookeri* digestive gland (DG, green curves), crops (C, purple curves), intestines (I, blue curves), and feces (F, red curves) after being fed with *P. crocata* (black curve). These profiles are compared according to their phenotype (MP = mineral, PBAS sample; OP = organic, MAS sample).

254-nm PDA chromatograms obtained from *P. crocata* and the biological compartments of *N. hookeri* fed on this species are collated in Figure 5.

**Figure 6:**
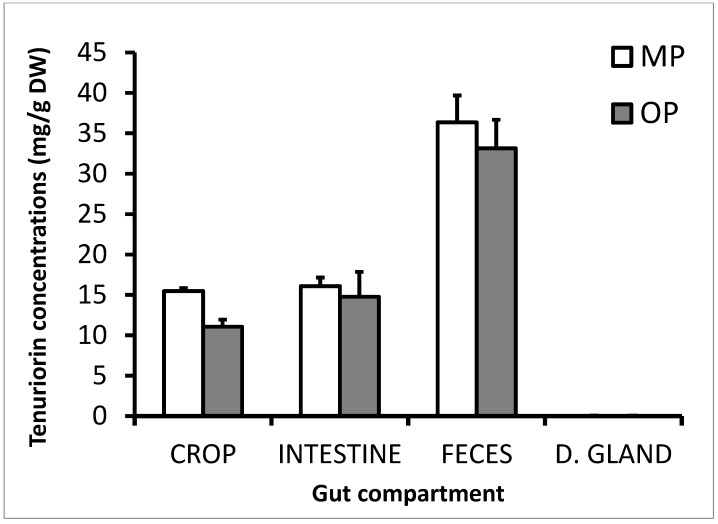
Levels of tenuiorin (means ± standard deviation) found in the snail gut compartments after the consumption of *P. crocata*, according to the snail phenotype (MP = Mineral and OP = Organic).

Stictic acid and its derivatives (i.e., constictic, cryptostictic, and norstictic acids) could not be identified in any digestive part of the snail nor its feces. Conversely, the tridepsides tenuiorin and 4-*O*-methylgyrophoric acid were found in the crops, intestines, and feces but remained quasi-undetected in the digestive glands (Figure 6).

Tenuiorin was not quantifiable in the digestive gland of the snail. Tenuiorin concentration was dependent on the gut compartments (ANOVA, F_1,18_ = 7.212, *p* = 0.015) and of the snail phenotype (ANOVA, F_1,18_ = 34.088, *p* < 0.001). It is noteworthy that the concentration of tenuiorin in the feces (34.76 ± 2.26 µg/mg) was two to three-fold that of its value within the crops and intestines of *N. hookeri* (Tuckey test, *p* < 0.001). It is also noteworthy that the ratio of 4-*O*-methylgyrophoric acid to tenuiorin increased along digestive tract of the snail. While the value of this ratio was estimated to a rough 15% in *P. crocata*, it reached values up to 79% in the biological samples (Figure S10). The pulvinic acid derivatives could only be detected from the feces of the snails. While the 254 nm PDA chromatogram does hardly evidence the excretion of these compounds, a 419 nm UV chromatogram better emphasizes the detection of pulvinic acid, calycin, and pulvinamide (Figure S11). In the lichen, the concentration of calycin displayed a mean value (±Sd) of 1.17 ± 0.37 mg/g DW (values ranging between 0.76 and 1.57 mg/g DW). In the feces, the mean value (± Sd) reached 2.09 ± 1.22 mg/g DW, which did not differ significantly from that found in the lichen (ANOVA, F_2,9_ = 0.776, *p* = 0.489).

HPLC-DAD-MS analyses revealed the occurrence of a signal at 30.9 min that did not appear in the acetone extract of *P. crocata*. This feature will be referred to as PcA.

### 2.3. Differences in Metabolite Profiling According to the Gut Compartment of Snails Fed P. crocata

**Figure 7:**
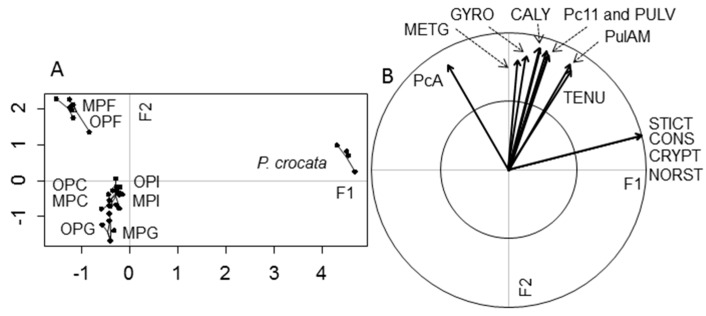
Graphs of the Partial Least Square-Discriminant Analysis (PLS-DA) performed on the specialized metabolites of the lichen *Pseudocyphellaria crocata*. (**A**) is the score plot obtained on the two first axes (F1, F2) that explain 94.50% of the constrained variances; (**B**) is the corresponding loading plots of specialized metabolites. The cross-model validation used for the discrimination of the lichen parts (8 components, 999 permutations, NMC = 0.668; *p* = 0.004) indicated statistically different biochemical profiles. The samples are crop (C), intestine (I), digestive gland (G), and feces (F) of organic (OP) and mineral (MP) phenotypes. The metabolites are constictic (CONS), cryptostictic (CRYPT), stictic (STICT), norstictic (NORST), pulvinic (PULV), gyrophoric (GYRO), and 4-*O*-methylgyrophoric (METG) acids, calycin (CALY), and pulvinamide (PulAM), Pc11 detected in the lichen but unidentified, and the unidentified PcA that only arose in the snail gut.

Twelve specialized metabolites were detected from *P. crocata* and gut compartments of snails fed upon this lichen. The first two-dimensional-axes of the PLS-DA extracted 94.50% of the constrained variance, the first axis (F1) accounting for 65.83% and the second axis (F2) for 28.67% (Figure 7). The specialized metabolites contributed significantly to discriminating the lichen and the gut compartments (ANOVA, F_8,27_ = 16.701, *p* = 0.001) and they explained 90.01% of the total variance (i.e., residual variance = 9.99). Constictic, cryptostictic, stictic, and norstictic acids were specific to *P. crocata*, as they were not detected in snail gut compartments, even in feces. Snail feces were grouped on the second axis, their metabolite composition being dominated by 4-*O*-methylgyrophoric acid and the unidentified compound PcA, not found elsewhere. The discrimination of the other snail compartments (crop, digestive gland, intestine) from snail feces was mostly due to either their low content or lack of lichen metabolites. Snail metabolism was quite similar for both phenotypes on *P. crocata*, a result indicated by the permutation test on the model with snail phenotype as explanatory variable (F_1,24_ = 1.607; *p* = 0.194). In this model, no interaction occurred between phenotype and gut compartment (F_3,24_ = 0.532; *p* = 0.765). Therefore, gut compartment was the only significant variable (F_3,24_ = 36.813; *p* = 0.001).

## 3. Discussion

### 3.1. Chemical Profile of Usnea taylorii and Toxicity of Lichen Metabolites

The extraction yield of *U. taylorii* displays a low value for a lichen species [21].

Usnic acid is a chemotaxonomic marker of the *Usnea* genus [22] (Figure S3). The determined concentration of usnic acid (0.4%) lies within the concentrations determined by Cansaran et al. in various *Usnea* species that encompassed a 0.2%–6.9% range of dry lichen weight [23].

Numerous investigations shed light on the ecological relevance of usnic acid describing its allelopathic and anti-feeding properties against plant-eating animals [24]. As to this latter point, usnic acid exhibited an acute toxicity against larvae of the polyphagous insect herbivore *Spodoptera littoralis* with a LD_50_ at 8.6 µM for (−)-usnic acid and 90.8 µM for (+)-usnic acid [25] as well as towards the larvae of the house mosquito *Culex pipiens* with a dose-dependent larval mortality [26]. Likewise, a recent study reported on the detrimental effects of usnic acid on the growth and survival rate of the larvae of the lichen-eating moth *Cleorodes lichenaria* [27]. Harmful effects of usnic acid were also demonstrated on vertebrate herbivores. During the winter of 2004, the poisoning and subsequent death of ~500 elks (*Cervus canadensis*) was reported in Wyoming and related to the consumption of *Xanthoparmelia chlorochroa* and more accurately to its usnic acid content [28]. On the opposite, reindeer and caribou survive on a winter diet consisting exclusively of lichens [29]. Such species might have acquired the capacity to handle toxic metabolites by a detoxification involving rumen microflora, which would house bacteria resistant to antibiotic secondary components [30].

**Figure 8:**
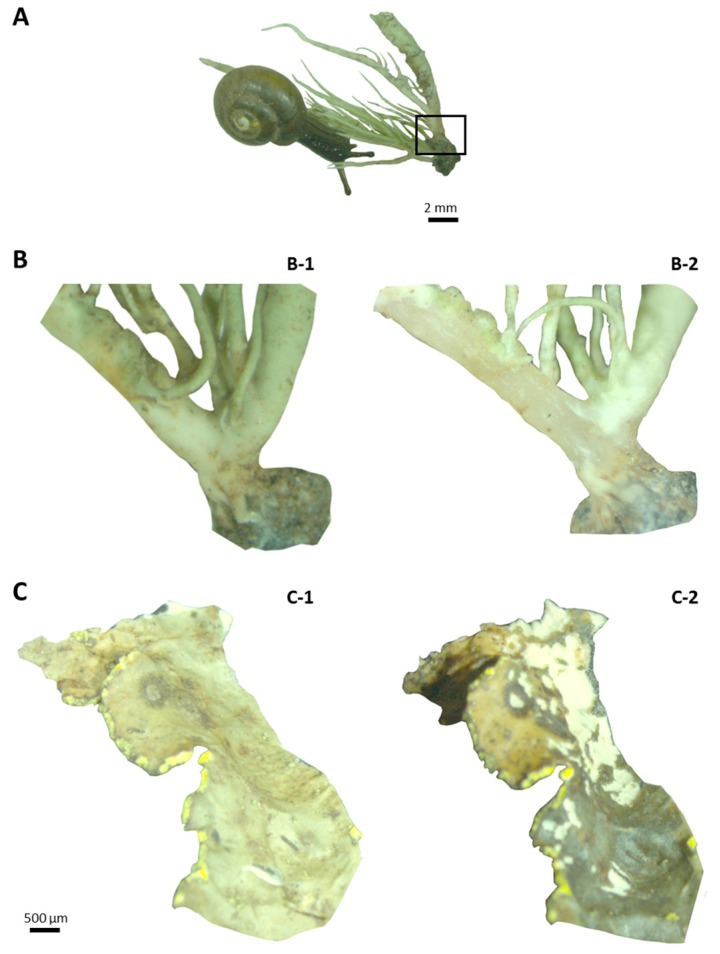
(**A**) *N. hookeri* consuming *U. taylorii* thallus. *U. taylorii* (**B**) and *P. crocata* (**C**) thalli before and after snail consumption. The medulla remained untouched for both lichens. The specimens were dry before consumption (**B-1** and **C-1**) and wet after snail feeding (**B-2** and **C-2**). The inset (**A**) corresponds to the magnification used in B

The role of bacteria in detoxification cannot be considered in snails because they are hindgut fermenters that harbor intestinal bacteria [31]. Although usnic acid could be detected in the different gut compartments, the concentration of this dibenzofuran derivative in the feces was shown to be about seven times as much as in the intestine. By comparison to the lichen, the OP-snail feces were enriched in usnic acid by a three-fold factor. It is assumed that focal environmental variations might favor adaptation to local conditions for nested populations such as *N. hookeri* that is restricted to focal habitats owing to its poor dispersal activity [15]. We hypothesized that the snail was able to sequestrate usnic acid in the digestive gland, since this organ is involved in the production of digestive enzymes [32], hydrogen production, nutrient absorption [33] and excretion [34]. Regarding this specific example, our hypothesis was invalidated because of the low levels of usnic acid found in the digestive gland (Figure S11). Therefore, it seems that snails usually feeding on *U. taylorii* might have acquired the ability to eliminate usnic acid by active transport. Conversely, fumarprotocetraric acid could not be detected from any gut compartment. The observation of the consumed *U. taylorii* thalli revealed that *N. hookeri* only fed on the superficial cortex of the lichen, while the bulk of fumarprotocetraric acid is contained in the medulla (Figure 8B) [35]. Therefore, this depsidone may not be ingested by *N. hookeri*, while usnic acid is consumed because of its accumulation in the cortical layers of the lichens [36]. One can also imagine that the minute amounts of fumarprotocetraric acid encountered in *U. taylorii* precluded its detection from the biological samples.

### 3.2. Toxicity of the Metabolites Detected in Pseudocyphellaria crocata and Strategies Implemented by *Notodiscus hookeri* to Cope with Lichen Metabolites

The chemical profile obtained for *P. crocata* is in line with previous reports describing the chemistry of this species [37,38,39,40]. As to the quantification of these major metabolites, Nybakken et al. described a comparable content in stictic acid (0.6%) but reported on a much higher tenuiorin concentration [41]. However, as lichen specialized metabolites participate in ecological interactions, their concentration is known to vary greatly based on environmental conditions [42].

The mass spectrometric signals of the specialized metabolites of *P. crocata* are rationalized in supporting material, being consistent with data published elsewhere (Figures S5–S9).

The compounds described from this lichen species are known to exert deterring effect and/or toxicity to phytophagic species. The repulsive effects of lichens whose chemistry is dominated by tenuiorin and methylgyrophoric acid derivatives were evidenced [5,6]. The deterrent effect of stictic acid was described towards the coleopteran *Lasioderma serricorne* [5] and against the generalist vertebrate *Myodes glareolus* [43]. Likewise, stictic and constictic acids occurring in *Lobaria pulmonaria* were shown to protect this lichen from being grazed [44]. Reducing the content of depsidones in *Lobaria pulmonaria* by acetone-rinsing caused a significant increase in mollusc grazing [45,46]. Based on their bright yellow color, the pulvinic acid derivatives are obviously ascribed to the soredia of *P. crocata*, i.e., symbiotic propagules occurring on the upper surface of the thallus as dust-like particles comprising a few algal and fungal cells. Such a spatial mapping refers to the optimal defense theory that predicts the allocation of the most efficient defensive compounds to the most valuable tissues for lichen’s fitness [47]. As to the specific example of *P. crocata*, Gauslaa evidenced that phytophages avoided to feed on soralia which might be significant for their survival and for the early establishment of vulnerable germinating soredia [44]. The anti-feeding properties of the closely related vulpinic acid are supported by its well-documented toxicity to several vertebrates [48]. Giez and co-workers also demonstrated the growth-retarding effects of calycin and vulpinic acid against the larvae of *Spodoptera littoralis*. Furthermore, the treatment of larvae with calycin resulted in an unusually high proportion of imagos with deformed wings [49].

In this study, PLS-DA revealed that the compounds described from *P. crocata* clustered into three main groups. The first one corresponded to depsidones. Despite being major metabolites of *P. crocata*, stictic acid derivatives could not be detected from any biological sample obtained from the snails fed with this lichen. As previously observed with *U. taylorii*, it appeared that the gastropods grazed the upper cortex and the photobiont layer whereas the medulla remained intact, leaving the underlying white medulla exposed, consistently with data published elsewhere on this lichen [3] (Figure 8C). As molecules of the stictic acid chemosyndrome (i.e., constictic, cryptostictic, stictic and norstictic acids) are known to display a medullary distribution [50,51], it is likely that *N. hookeri* did not ingest these compounds. However, the feeding pattern of the snail might not necessarily reflect a deterring effect of these depsidones. Indeed, for phytophagous grazers one challenge is to gain enough dietary nitrogen in a carbohydrate-rich diet. Therefore, the photobiont layer should represent the most attractive tissue to lichenivores as it displays the highest nitrogen content in the lichen thallus [52]. This is especially true for cyanolichens as *P. crocata* that can fix atmospheric N_2_ thanks to their cyanobacterial biont generally resulting in high N concentration [48].

All the other compounds described from *P. crocata* display a cortical distribution: metabolites of the gyrophoric acid series [53] and pulvinic acid derivatives [54,55] suggesting that they might have been ingested by the snail given the observed grazing pattern. A second group of compounds was plotted between the lichen and the feces groups. These metabolites were the tridepsides (tenuiorin, 4-*O*-methylgyrophoric acid and gyrophoric acid), the pulvinic acid derivatives (pulvinic acid, pulvinamide and calycin) and the unidentified compound Pc11. Most of these molecules were excreted in slightly lower concentration compared to that obtained from the lichen itself, except for 4-*O*-methylgyrophoric acid which tended to be in a slightly higher concentration in the feces instead. The compared chemical profiles of lichens and snails samples revealed a concomitant diminution of the signals associated to tenuiorin with an increase of the peaks associated to 4-*O*-methylgyrophoric acid, suggesting the conversion of the former into the latter. This assumption is further strengthened by the particularly reduced yield of excretion of tenuiorin compared to the other metabolites of this group on the PLS-DA plot. The ester bond of depsides is known to be easily hydrolyzed under a variety of physicochemical circumstances. Likewise, the hydrolysis of methyl esters of lichen depsides to afford the corresponding carboxylic acid was described to occur in a variety of experimental conditions [56]. Due to the easiness of these chemical conversions, it appears more likely that such transformations rely on physicochemical conditions rather than involving specific enzymatic systems. As such, the slightly basic pH of the crop (which we estimated to be around 8.0, maybe due to salivary glands secretions) might be sufficient to trigger the alkaline hydrolyses described herein [57].

As to pulvinic acid derivatives, the yellow color of the feces produced by the group of *Notodiscus hookeri* fed with *P. crocata* indicated that the gastropod also consumed the soredia of the lichen. This is an interesting outcome as previous literature reported that snails avoided the well-defended bright yellow soralia that accumulate the pulvinic acid derivatives [44]. This highly localized concentration of calycin shall provide high levels of defense in early stages of juvenile thalli and support the optimal defense theory [47]. The partitioning of pulvinic acid derivatives within the thallus of *Pseudocyphellaria crocata* might be considered as a further example of optimal defense theory and suggests that *N. hookeri* developed strategies to overcome the toxicity of this suite of metabolites. The data described herein support this assumption as neither calycin, pulvinic acid, nor pulvinamide could be evidenced from the gut compartments of the snails, being only detected in its feces. For this second group of compounds, it seems that *N. hookeri* ingests these metabolites without absorbing them, circumventing the poisoning that might be induced by these lichen substances.

At last, PcA only arose in the feces of the snail, being absent from all other digestive compartments and also from *P. crocata*. This is consistent with the hypothesis that PcA might represent a metabolized lichen molecule. Nevertheless, this compound did not ionize under the mass spectrometric settings used in this study and the paucity of the biological material precluded the isolation of this metabolite for structural elucidation purposes.

## 4. Materials and Methods

### 4.1. Snail Collection and Lichen Material

Adult individuals of *N. hookeri* were collected on Possession Island during 2015 austral summer (Figure S1). Habitat typology, soil composition and topography have been previously described [15]. Snails came from two coastal plains, Baie Américaine (BUS, 46°23′26.16″ S; 51°48′8.31″ E, 30 m) and Pointe Basse (PBAS, 46°22′5.48″ S; 51°43′26.39″ E, 100 m) making up the mineral phenotype (MP) group and from two fell-fields, Crête de l′Alouette (ALOU, 46°22′55.21″ S; 51°47′20.35″ E, 300 m) and Mascarin Summit (MAS, 46°26′10.09″ S; 51°45′20.58″ E, 600 m) constituting the organic phenotype (OP) group. Four hundred snails (100 × 4 sites) were brought back to mainland France on 8 December 2015. From this date until 15 January 2016, all individuals were fed with the lichen *Orceolina kerguelensis*, an opportunistic chlorolichen found everywhere in the island.

*U. taylorii* and *P. crocata* were harvested on Possession Island during 2015 austral summer and used as food in our feeding experiment. The foliose cyanolichen *P. crocata* occurred at PBAS where the snail was collected and the fruticose chlorolichen *U. taylorii* was encountered at MAS in the snail habitat. The former lichen specie was found in grassy coastline dominated by Poaceae (*Poa cookii*, *Agrostis magellanica*) and fernbrakes (*Blechnum penna-marina*) while the latter only occurred in windy and rocky areas, deprived of vegetation except lichens [15]. Both these lichens were identified by Dr. Damien Ertz (Botanic Garden, Meise, Belgium). Voucher specimens were deposited at the herbarium of the Faculty of Pharmacy of Rennes 1, Department of Pharmacognosy and Mycology, under the respective references REN000147 and REN000148.

### 4.2. Feeding Experiment

Forty snails from BUS, PBAS, ALOU, and MAS were divided into two subgroups and each of them was fed either with *P. crocata* or *U. taylorii*. The snails had similar sizes and weights, except those from the MAS site, which were significantly bigger and heavier (Table S1, Supplementary Materials). The snails were reared by 20 in plastic boxes (7.5 × 6 × 4.5 cm) with a moist sterile gauze in the bottom. The boxes were held in a climatic chamber under a light/dark photoperiod set as follows: 10 °C day (16 h) and 6 °C night (8 h). To circumvent microbial infections, the boxes were washed regularly and sterile water was poured over them every two days to keep an appropriate level of humidity. After two months, the snails were frozen at −20 °C prior to being carefully dissected under stereomicroscope (Stemi 2000C, Zeiss, France) Three gut compartments were considered: crop (Cr), digestive gland (DG), and intestine (In) Figure S2. The feces (Fe, i.e., rectum contents) were harvested once a week and kept at −20 °C prior to being lyophilized and extracted as described in the chemical section.

### 4.3. Chemistry

#### 4.3.1. Extraction

Each gastropod was carefully dissected into three parts: crop, digestive gland, and intestine. The dissected tissues and the feces were separately extracted with acetone (3 × 0.5 mL, 20 min. each) and allowed to dry at room temperature.

Four pieces of *Usnea taylorii* (0.5 to 1.0 g) were extracted three times with acetone (5 mL, 20 min) at room temperature and were air-dried until the solvent had evaporated. The extraction yields ranged between 0.4% and 1.0% of dry mass of lichen material for a mean value of 0.8% ± 0.2%.

Four pieces of *Pseudocyphellaria crocata* (0.5 to 2.0 g) were also subjected to the procedure described from *U. taylorii* The extraction yields ranged between 3.7% and 6.5% of dry mass of lichen material for a mean value of 5.3 ± 1.2%.

#### 4.3.2. HPLC-DAD-MS Analyses

##### Preparation of the Samples

The samples corresponding to crop, intestine, digestive gland and feces extracts were prepared at a concentration of 0.50 mg/mL in distilled tetrahydrofuran (THF). Lichen extracts were resuspended at a concentration of 0.50 mg/mL (*P. crocata*) or 0.25 mg/mL (*U. taylorii*) in THF.

##### Instrumental Settings

The extracts were filtered through 0.45 mm disposable filters prior to injecting a 10 µL aliquot to the HPLC-DAD-MS device. The LC-DAD-ESI-MS analyses were performed on a Prominence Shimadzu HPLC system (Marne La Vallée, France) equipped with a Kinetex C_18_ HPLC column (100 × 4.6 mm, 2.6 µm, 6A, Phenomenex) and consisting of a quaternary pump (LC20ASDP), a surveyor autosampler (SIL-20AHT), and a diode array detector (SPD-M20A). The separation was achieved using an acidic water/acetonitrile system. The gradient of the mobile was as follows: A (0.1% formic acid in water) and B (0.1% formic acid in acetonitrile); T 0–5 min, 20% B linear; 5–30 min, 80% B linear; 30–35 min, 100% B linear; 35–42 min, 100% B; 42–45 min, 20% B linear; 45–48 min, 20% B. The flow rate was 500 µL/min. The ESI-mass spectra were obtained from an Expression Advion CMS apparatus (Advion, Ithaca, NY, USA). The mass spectra were recorded in the negative ion-mode in a mass range of 100 to 1200 Da, applying the following parameters: detector gain 1200, ESI voltage 3.5 kV, capillary voltage 180 V, source voltage 20 V, source voltage dynamic 20 V, nebulizer gas pressure 60 psig, desolvation gas flow rate 4 L/min, capillary gas temperature 250 °C and source gas temperature 50 °C. Usnic acid was detected and quantified at 290 nm, tenuiorin and stictic acid at 254 nm and calycin at 419 nm [20].

### 4.4. Statistical Analyses

As to the main metabolites found in gut compartments (i.e., usnic acid, tenuiorin, and calycin), the calibration curves allowed to quantify these metabolites in the different samples. *T*-tests (one factor) or ANOVAs (two factors) followed by *post-hoc* Tukey tests were used to compare the values between snail ecophenotypes and between lichen and gut compartments.

To compare the differences in metabolite profiling according to the gut compartment (including feces) and between *P. crocata* and gut compartments, a Partial Least Square-Discriminant Analysis (PLS-DA) [58] was conducted on the normalized values of areas of specialized metabolites. Prior to PLS-DA, we proceeded to fourth-root transformation, centering, and autoscaling of the values, because null values and great differences in concentrations according to metabolite profiles were observed [59]. A cross-model validation was applied to the PLS-DA to check for significant discrimination (function “MVA.cmv” and “MVA.test”, package “RVaidememoire”) [60]. The percentages of constrained and residual variances were obtained by a redundancy analysis (RDA) on the transformed values. A second RDA analysis considered gut compartments, snail phenotype, and site as explanatory variables to answer whether each of these variables was significant in the model. All statistical analyses were made using R software V. 3.3.1 (Vienna, Austria) [61].

## 5. Conclusions

As an exclusive lichen-feeder, *Notodiscus hookeri* depends on a trophic resource containing several series of toxic metabolites, especially towards invertebrates. *N. hookeri* appears as a generalist lichen feeder able to consume toxic metabolite-containing lichens, irrespective of their occurrence in its focal habitat and independently of the ecophenotype. The snail metabolism seems to be based on four non-exclusive processes depending on the considered metabolite: (1) avoidance of potentially harmful lichen metabolites by avoidance of the lichen medulla; (2) passive transport of pulvinic acid derivatives through the digestive tract of the gastropod, resulting in feces containing a roughly similar concentration of metabolites compared to the lichen; (3) hydrolysis of esterified tridepsides, most likely due to gut lumen alkalinity; and (4) excretion of some compounds in higher yields than those present in the resource. Regarding this latter strategy, the specific example of usnic acid from *Usnea taylorii* suggests that this gastropod implemented an active extraction strategy to overcome potential intoxications. This versatility most likely accounts for the widespread distribution of this gastropod on Possession Island.

## Supplementary Materials

Supplementary materials are available online.
