# Supplementary material for: Which Specialized Metabolites Does the Native Subantarctic Gastropod Notodiscus hookeri Extract from the Consumption of the Lichens Usnea taylorii and Pseudocyphellaria crocata?
Source: Molecules. 2017 Mar 8;22(3):425. doi: 10.3390/molecules22030425 (PMC6155395; doi:10.3390/molecules22030425)
Supplement: Supplementary file 1 [file molecules-22-00425-s001.pdf]

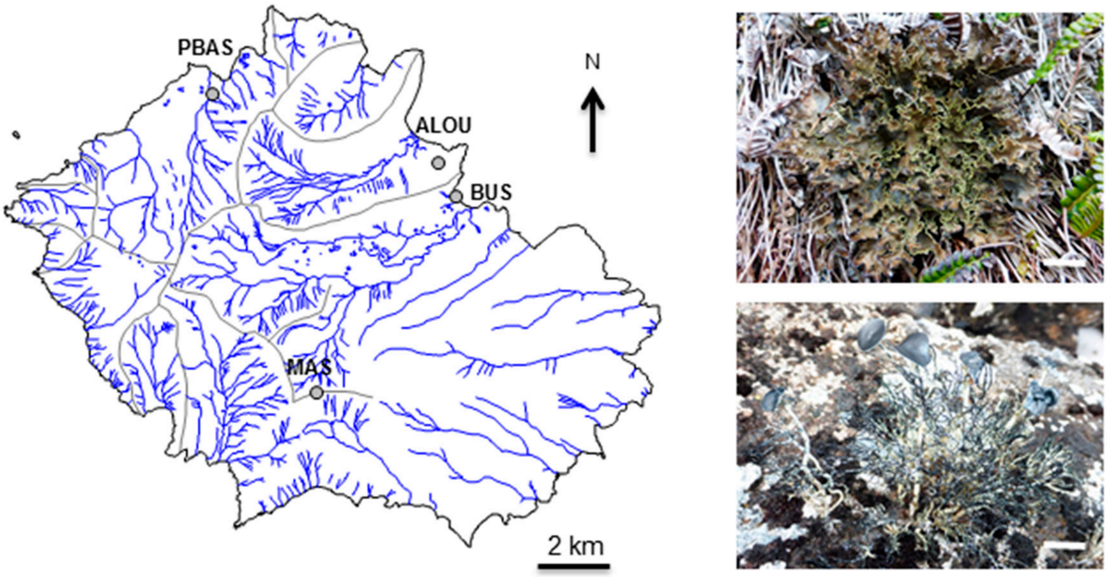

Figure S1: Study sites in Possession Island that belongs to Crozet Archipelago (45° 30′ – 46° 30′ S; 50° 00′ – 52° 30′ E) in the sub-Antarctic region. Snails were sampled Baie américaine (BUS) and Pointe Basse (PBAS) for the mineral phenotype and organic phenotype were collected at Crête de l'Alouette (ALOU) and Mascarin (MAS) . *Pseudocyphellaria crocata* (upper photograph) occurred at Pointe Basse (PBAS) and *Usnea taylorii* (lower picture) was present at Mascarin (MAS). Photographs courtesy of Damien Ertz (Botanic Garden, Meise, Belgium). Scale bars on lichen photographs correspond to 1 cm.

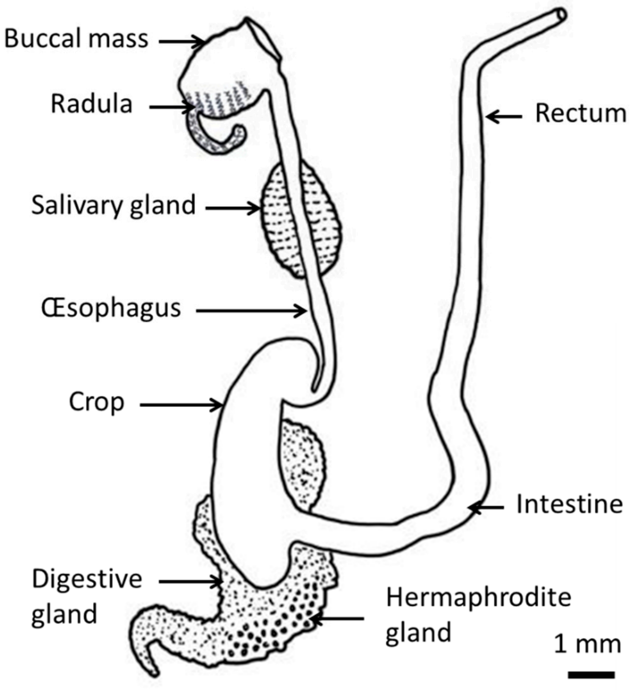

Figure S2: The digestive tract of *Notodiscus hookeri*. Gut compartments extracted after the feeding experiment were the crop, the digestive gland, the intestine and the rectum content (*i.e.* feces).

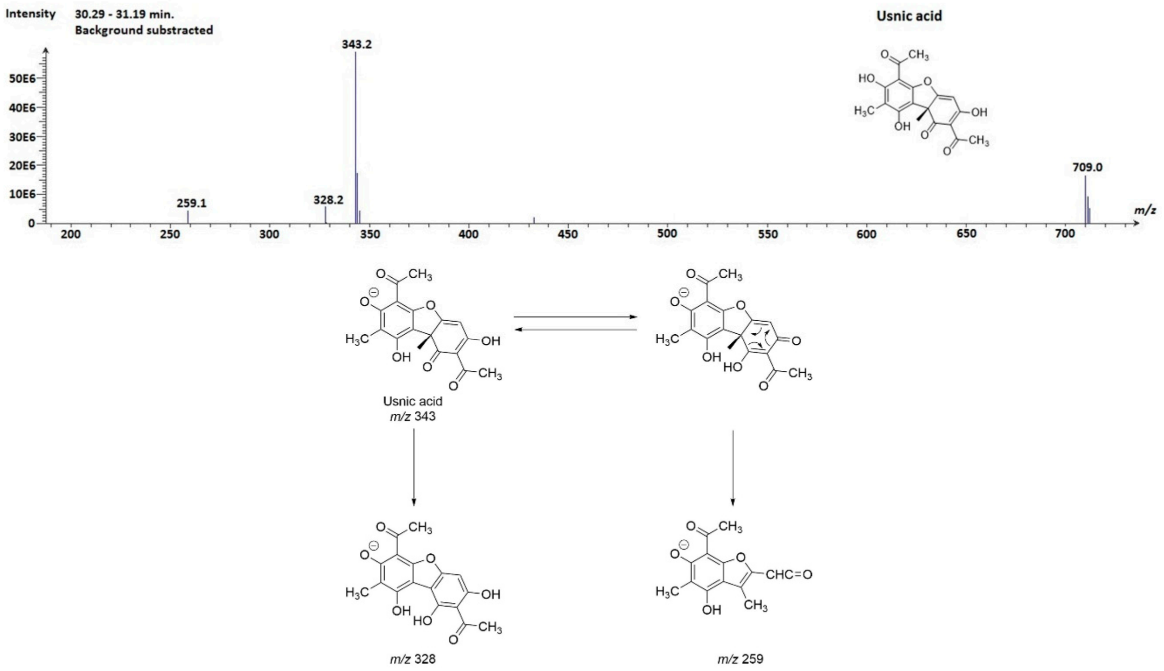

Figure S3: NI ESI mass spectrum of usnic acid and proposed fragmentation pathway

The mass spectrum of usnic acid reveals a fragment ion at *m*/*z* 328 that stems from the loss of a methyl radical, the driving force of which is the aromatization of cycle C to yield an energetically favoured dibenzofuran core. The formation of *m*/*z* 259 might then be explained by a retro Diels-Alder process as illustrated in Figure S3, consistently with previous reports [1,2].

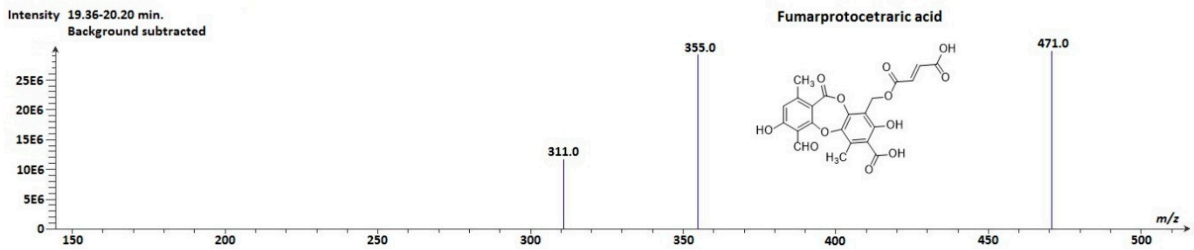

Figure S4: NI-ESI mass spectrum of fumarprotocetraric acid.

The loss of the fumaric acid moiety to afford *m*/*z* 355 from this depsidone side chain is consistent with previous reports [3]

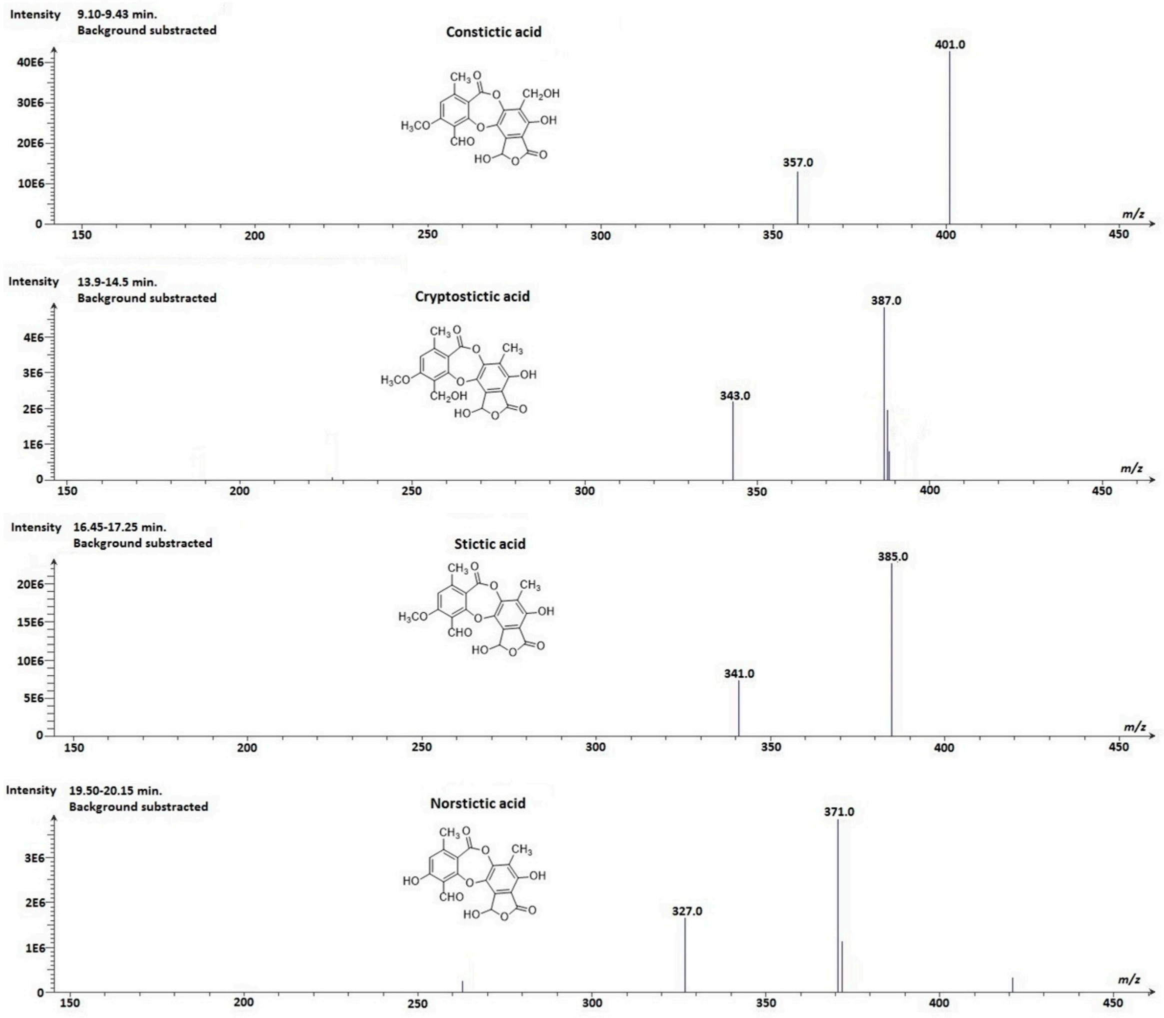

Figure S5: NI-ESI mass spectrum of depsidones identified in *P. crocata*

All depsidones revealed a prevalent deprotonated molecule accompanied by a fragment displaying a loss of 44 mass units. This neutral loss of CO_2 on the central core of the depsidone scaffold is presumed to afford a dibenzofuran moiety [4]. The structural assignments of these depsidones are further supported by the order of elution of these metabolites as displayed in the main text [4,5].

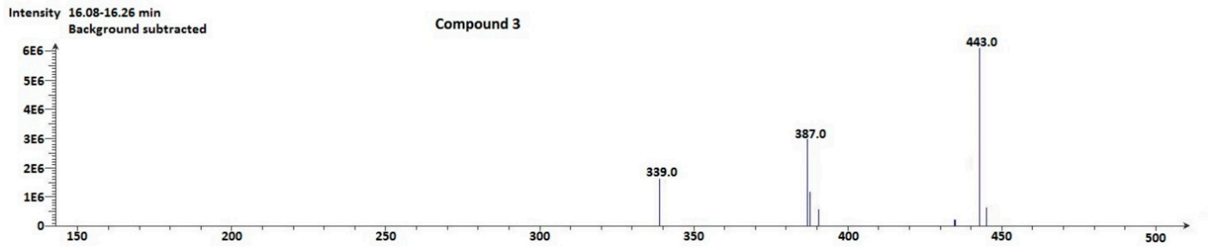

Figure S6: NI-ESI mass spectrum of compound **3** (Pc3) identified in *P. crocata* (retention time 16.08-16.26 min).

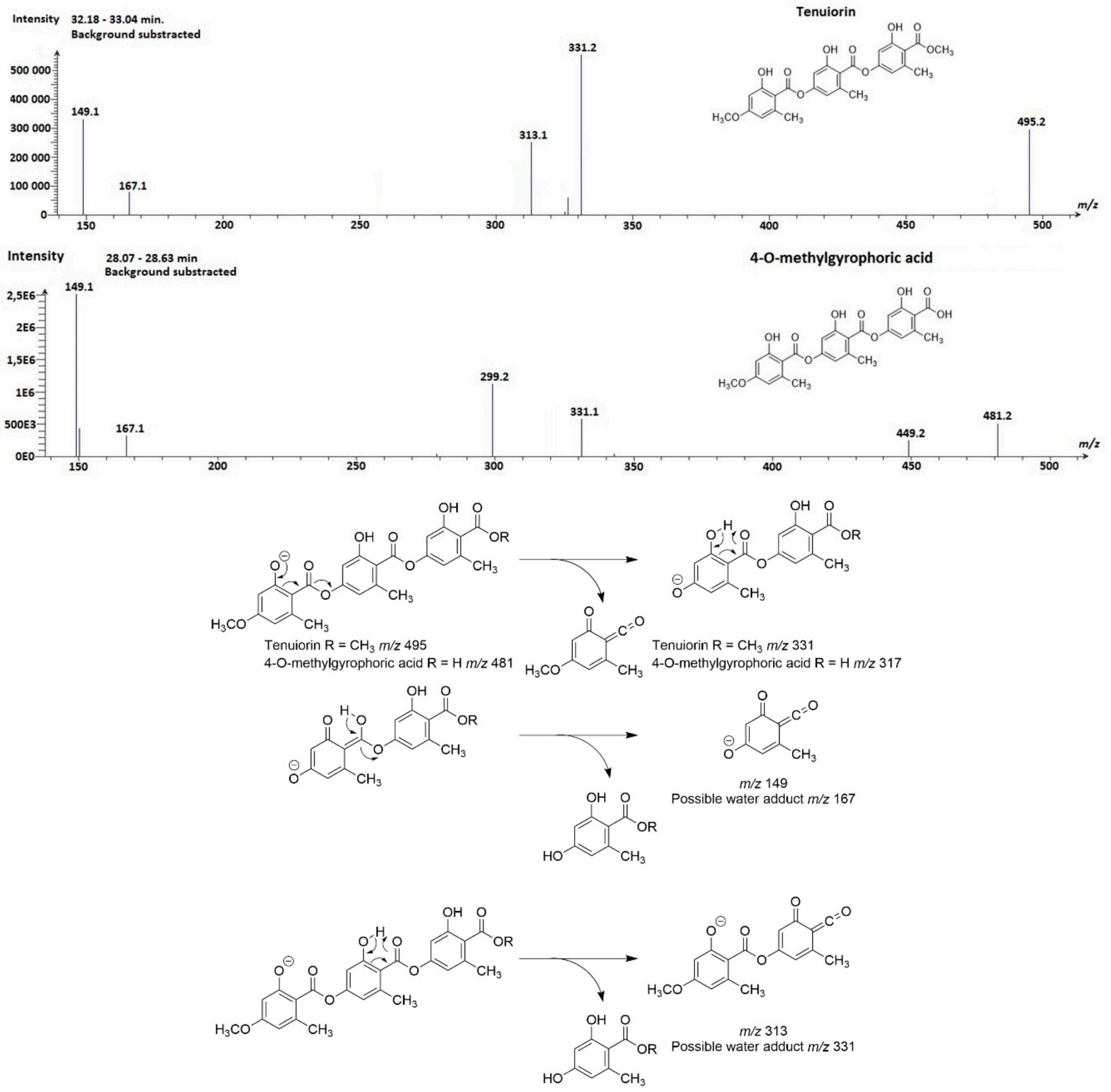

Figure S7: NI-ESI mass spectra of tenuiorin and 4-O-methylgyrophoric acid in NI-ESI-MS and proposed dissociation pathways. Note that the sites bearing the charges are arbitrary.

A first mechanism involves an electron delocalization from the phenoxyl anion followed by consecutive aromatic unit losses. A second pathway is initiated by McLafferty type rearrangements with subsequent acyl cleavages. As to tenuiorin, such fragmentation patterns account for *m/z* 331, 313 and 149. The interpretation of *m/z* 167 turned out to be difficult as it cannot derive from the $[M-H]^-$ ion by any established fragmentation mechanism. However, the fact that *m/z* 167 is 18 Da greater than *m/z* 149 seems to hint that the former might have one more H_2O molecule than the latter. A likely assumption regarding this signal is that it might stem from gas phase ion-molecule reactions resulting in water adducts [6]. The highly reactive ketene moiety has shown a tendency to covalently attach water molecules to yield the corresponding carboxylic acid [7-9]. Such a gas phase reactivity was suggested for the lichen depside perlatolic acid [10]. Likewise, it can be proposed that *m/z* 331 might indeed correspond to an H_2O adduct of *m/z* 313 rather than being the genuine fragment ion displayed in Figure S7. This alternative fragmentation hypothesis is further strengthened by the arising of *m/z* 331 in the spectrum of 4-O-methylgyrophoric acid which can be explained by no logical neutral loss whereas *m/z* 313 can.

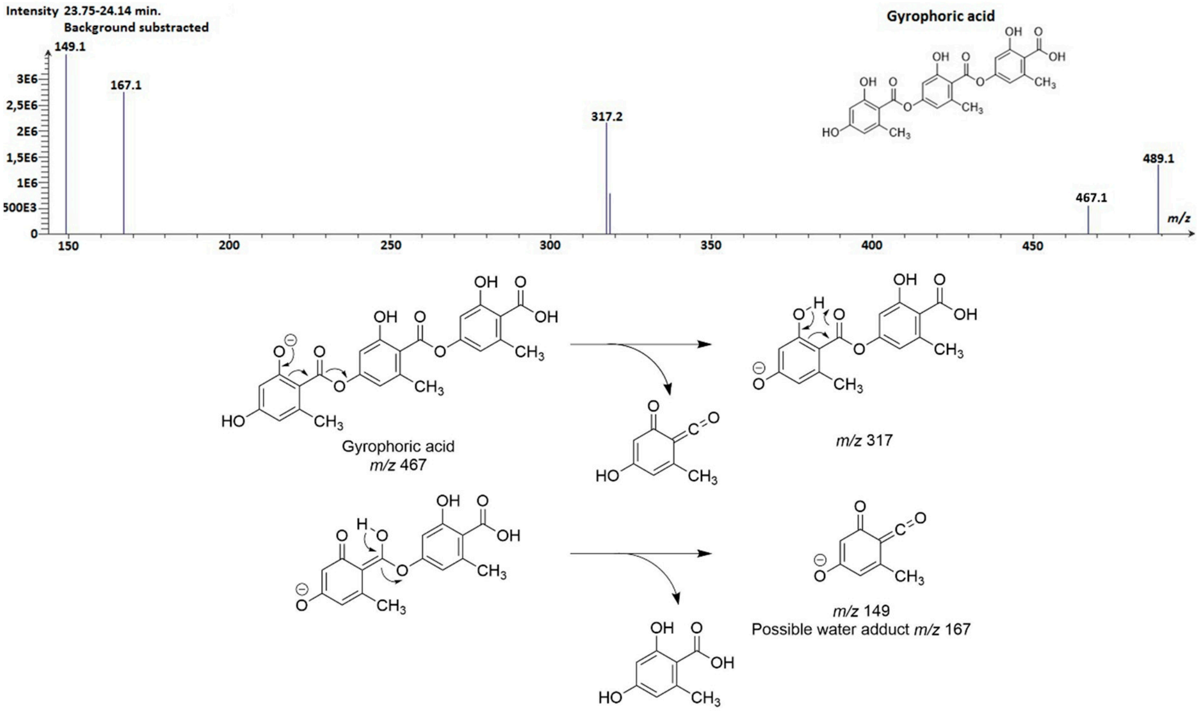

Figure S8: NI-ESI mass spectrum of gyrophoric acid and proposed dissociation scheme. The site bearing the charge is arbitrary.

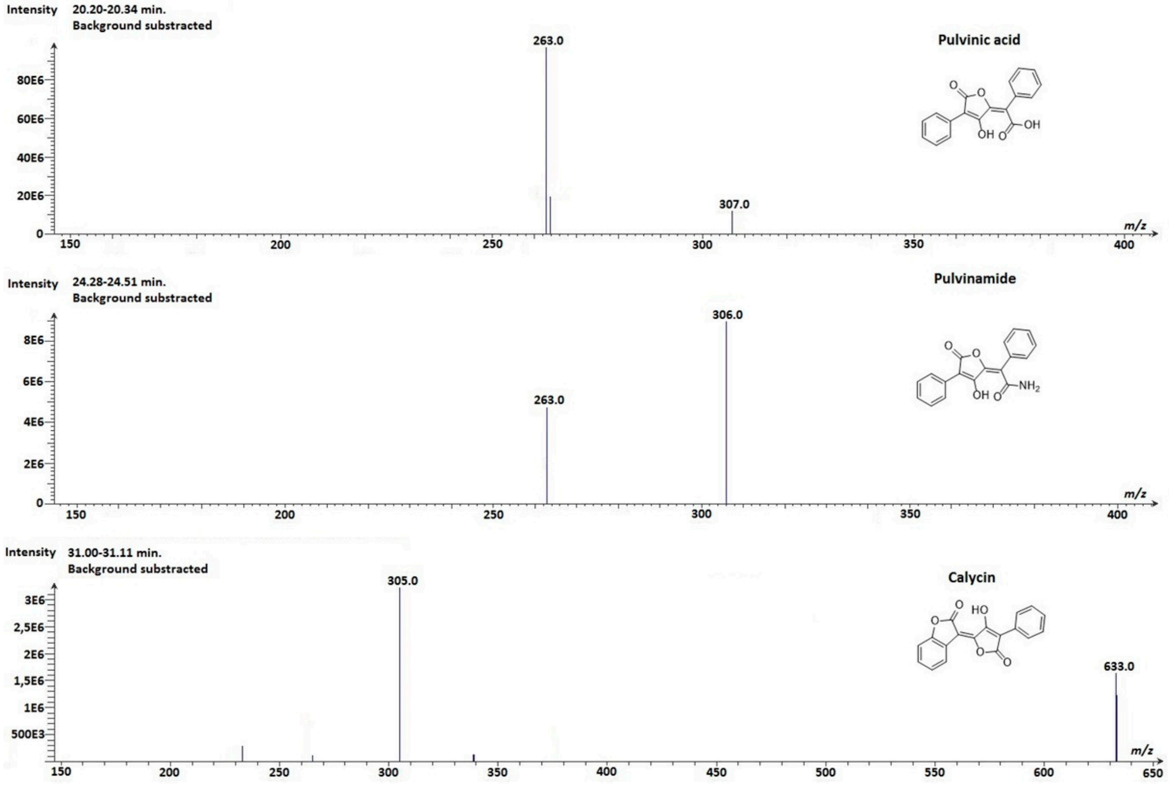

Figure S9: NI-ESI mass spectrum of pulvinic acid derivatives identified in *P. crocata*

Pulvinic acid and pulvinamide revealed a common fragment at *m*/*z* 263, respectively through the loss of their carboxylic acid and amide groups. In the mass spectrum of calycin, *m*/*z* 633 stands for a dimerized sodium adduct.

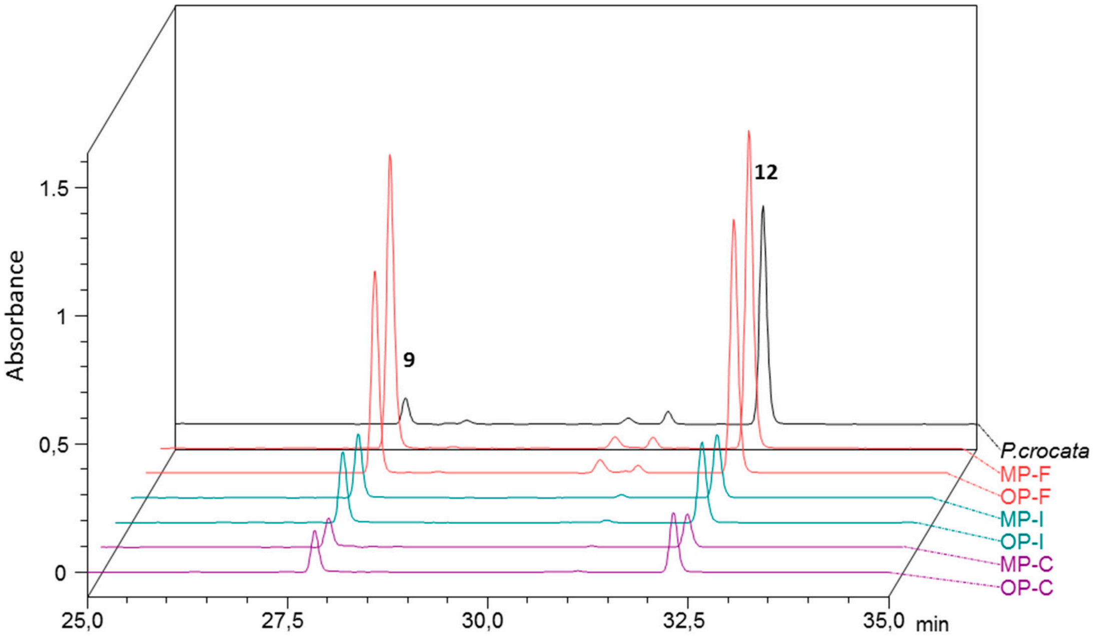

Figure S10: Evolution of the ratio of 4-O-metylgyrophoric acid (9) and tenuiorin (12) in *P. crocata* (black curve) and along the digestive tract of *N. hookeri:* crops (C, purple curves), intestines (I, blue curves) and feces (F, red curves). These profiles are compared according to their phenotype (MP = mineral, PBAS sample; OP = organic, MAS sample).

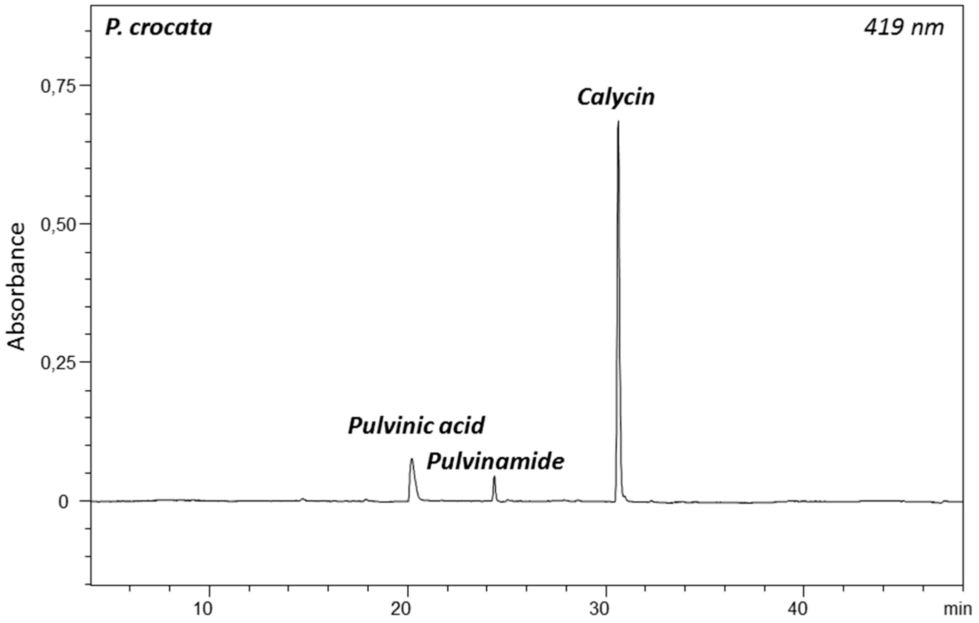

Figure S11: PDA-chromatogram at 419 nm of the acetone extract of *P. crocata* highlighting its pulvinic acid derivatives content.

Table S1: Measurements made on the snails *Notodiscus hookeri*, fed on *Usnea taylorii* or *Pseudocyphellaria crocata*. Initial shell sizes in mm (A) and fresh weights in mg (B) were represented by their mean values (± Standard deviation). The two-way ANOVA values are given for the factor Site and the factor Box. With both variables, the interaction being not significant, the result was not shown. The lowercase superscript letters a and b indicate significant differences between MAS and the three other sites.

A-Shell sizes	BUS[a]	PBAS[a]	ALOU[a]	MAS[b]	Anova
Box *U. taylorii*	4.38 ± 0.41	4.16 ± 0.68	4.24 ± 0.39	5.56 ± 0.57	Factor = Box;
Box *P. crocata*	4.31 ± 0.48	4.14 ± 0.68	4.29 ± 0.34	5.57 ± 0.52	$F_{1,152}$ =0.01, p=0.92
Anova	Factor = Site; $F_{3,152}$ = 64.2, p<0.001				

B-Fresh weights	BUS[a]	PBAS[a]	ALOU[a]	MAS[b]	Anova
Box *U. taylorii*	25.52 ± 6.43	19.83 ± 9.24	17.80 ± 6.23	41.62 ± 11.26	Factor = Box;
Box *P. crocata*	25.21 ± 7.34	19.28 ± 8.78	18.97 ± 5.91	41.36 ± 10.96	$F_{1,152}$<0.01, p=0.99
Anova	Factor = Site; $F_{3,152}$ = 60.6, p<0.001				

References

1. Huneck, S.; Schmidt, J. Lichen substances—126 mass spectroscopy of natural products—10. Comparative positive and negative ion mass spectroscopy of usnic acid and related compounds. *Biol. Mass Spectrom.* **1980**, *7*, 301–308.

2. Le Pogam, P.; Le Lamer, A.-C.; Legouin, B.; Boustie, J.; Rondeau, D. *In situ* DART-MS as a Versatile and Rapid Dereplication Tool in Lichenology: Chemical Fingerprinting of *Ophioparma ventosa*. *Phytochem. Anal.* **2016**, *27*, 354–363.

3. Holzmann, G.; Leuckert, C. Applications of negative fast atom bombardment and MS/MS to screening of lichen compounds. *Phytochemistry* **1990**, *29*, 2277–2283.

4. Parrot, D.; Jan, S.; Baert, N.; Guyot, S.; Tomasi, S. Comparative metabolite profiling and chemical study of *Ramalina siliquosa* complex using LC–ESI-MS/MS approach. *Phytochemistry* **2013**, *89*, 114–124.

5. Yoshimura, I.; Kinoshita, Y.; Yamamoto, Y.; Huneck, S.; Yamada, Y. Analysis of secondary metabolites from lichen by high performance liquid chromatography with a photodiode array detector. *Phytochem. Anal.* **1994**, *5*, 197–205.

6. Attygalle, A. B.; Kharbatia, N.; Bialecki, J.; Ruzicka, J.; Svatoš, A.; Stauber, E. J. An unexpected ion-molecule adduct in negative-ion collision-induced decomposition ion-trap mass spectra of halogenated benzoic acids. *Rapid Commun. Mass Spectrom.* **2006**, *20*, 2265–2270.

7. Sultan, J. Collision induced dissociation of deprotonated guanine: Fragmentation of pyrimidine ring and water adduct formation. *Int. J. Mass Spectrom.* **2008**, *273*, 58–68.

8. Neta, P.; Farahani, M.; Simón-Manso, Y.; Liang, Y.; Yang, X.; Stein, S. E. Unexpected peaks in tandem mass spectra due to reaction of product ions with residual water in mass spectrometer collision cells. *Rapid Commun. Mass Spectrom.* **2014**, *28*, 2645–2660.

9. Alechaga, É.; Moyano, E.; Galceran, M. T. Ion-molecule adduct formation in tandem mass spectrometry. *Anal. Bioanal. Chem.* **2016**, *408*, 1269–1277.

10. Guan, Z.; Liesch, J. M. Solvation of acylium fragment ions in electrospray ionization quadrupole ion trap and Fourier transform ion cyclotron resonance mass spectrometry. *J. Mass Spectrom.* **2001**, *36*, 264–276.
